# Investigation of the Effects of Ski Ergometer-Based Training on Respiratory Functions and Isokinetic Muscle Strength in Cross-Country Skiers

**DOI:** 10.3390/medicina62030543

**Published:** 2026-03-14

**Authors:** Buket Sevindik Aktaş, Esedullah Akaras, Muhammet Polat, Sıla Kara, Mine Kılıç

**Affiliations:** 1 Faculty of Sport Sciences, Erzurum Technical University, Erzurum 25050, Turkey; 2 Department of Physiotherapy and Rehabilitation, Faculty of Health Sciences, Erzurum Technical University, Erzurum 25050, Turkey; esedullah.akaras@erzurum.edu.tr; 3 Department of Physical Education and Sports, Institute of Winter Sports and Sports Sciences, Atatürk University, Erzurum 25240, Turkey; muhammetpolat0507@gmail.com; 4 Department of Physical Education and Sports, Institute of Health Sciences, Erzurum Technical University, Erzurum 25050, Turkey; silakara21@gmail.com (S.K.); minaklc01@gmail.com (M.K.)

**Keywords:** winter sports, cross-country skiing, isokinetic strength, respiratory functions, ski-erg training

## Abstract

*Background and Objectives*: Cross-country skiing requires high levels of upper-body strength and efficient respiratory function to sustain performance during sport-specific movements. This study aimed to examine the effects of an eight-week ski ergometer-based training program on upper-extremity isokinetic muscle strength and pulmonary function in competitive cross-country skiers. *Materials and Methods*: A total of 20 cross-country skiers voluntarily participated in the study (experimental group: *n* = 10, control group: *n* = 10). The research was conducted using a quasi-experimental controlled design. During the eight-week training period, the experimental group performed ski ergometer training three times per week at an intensity of 80–90% of maximal heart rate, with a target distance of 2.5 km per session, in addition to their regular training program. Measurements were obtained before and after the intervention. *Results:* Following the ski ergometer training period, significant increases were observed in FVC (F = 18.565, *p* < 0.001, ηp^2^ = 0.508) and FEV_1_ (F = 8.789, *p* = 0.008, ηp^2^ = 0.328), which were associated with enhanced respiratory muscle endurance and ventilatory capacity. Regarding the isokinetic strength parameters, the DPPE60 variable showed significant main effects of time (F = 33.770, *p* < 0.001, ηp^2^ = 0.652) and time × group interaction (F = 18.590, *p* < 0.001, ηp^2^ = 0.508), indicating higher upper-extremity strength values across the measurement period. Additionally, strong positive correlations were found between dominant and nondominant limbs (r = 0.79–0.92; *p* < 0.05), indicating balanced bilateral strength development and high neuromuscular coordination. *Conclusions*: Ski ergometer-based training was associated with improvements in upper-extremity peak power (DPPE60) and ventilatory capacity (FVC) beyond general training-related adaptations. These findings suggest that SkiErg training may be a useful complementary method for enhancing selected performance-related physiological parameters in cross-country skiers.

## 1. Introduction

Cross-country skiing is a high-intensity endurance sport that significantly enhances cardiovascular endurance, muscle strength, and respiratory capacity [1]. In this sport, in addition to traditional training methods, specific exercise protocols and technologically advanced training devices are used to enhance performance. In recent years, ski ergometers have become widely adopted tools in the training routines of cross-country skiers, and their effects on athletes’ physiological and biomechanical adaptations have been increasingly investigated [2,3].

Respiratory functions are considered an important physical fitness parameter, reflecting overall health status, and are closely related to muscle strength [4]. Several studies have demonstrated that respiratory functions have a significant impact on athletic performance [5,6]. It is known that, during normal breathing, skeletal muscles do not directly contribute to respiration; however, as exercise intensity increases, trunk and limb muscles assist the respiratory process [7,8]. Muscle strength is a critical component of physical fitness that influences both daily functional activities and athletic performance [9]. Moreover, insufficient muscle strength can lead to muscular imbalances and compensatory movement patterns, thereby increasing the risk of sports-related injuries [10]. In endurance-demanding sports such as cross-country skiing, isokinetic muscle strength plays a vital role in sustaining performance [10].

Ski ergometer-based training aims to enhance muscle strength and endurance by targeting the upper extremity and core muscle groups of cross-country skiers. Such training programs may have potential implications for injury prevention strategies and return-to-play protocols, although these outcomes were not directly examined in the present study. In addition, the use of ski ergometers holds significant potential in rehabilitation techniques for winter sports injuries, as it allows for controlled loading and facilitates functional recovery following musculoskeletal injuries commonly observed in winter sports. In this context, examining the effects of ski ergometer training on respiratory function and isokinetic muscle strength may help clarify mechanisms underlying performance enhancement and safe return to sport [10]. Upper-body strength, in particular, plays a critical role in achieving high performance in cross-country skiing, and resistance-based training tools such as the ski ergometer are highly effective in strengthening these muscle groups [11,12]. To our knowledge, no previous study has simultaneously examined upper-extremity isokinetic strength and respiratory function following SkiErg-based training in competitive cross-country skiers.

Ski ergometer-based training has been increasingly used in the preparation of cross-country skiers; however, evidence regarding its combined effects on upper-extremity isokinetic strength and respiratory function remains limited. The primary aim of this study was to examine the association between an eight-week ski ergometer-based training program and changes in upper-extremity isokinetic muscle strength and respiratory function parameters in cross-country skiers. It was hypothesized that ski ergometer-based training would be associated with improvements in DPPE60, FVC, and FEV_1_ parameters. The findings of this study are expected to contribute to the scientific understanding of training adaptations in cross-country skiers.

## 2. Materials and Methods

### 2.1. Research Design

The present study employed a quasi-experimental controlled pre–post design. Random allocation of participants was not feasible because of the existing team structure and training organization. Participants were therefore assigned to the experimental and control groups based on team affiliation and coaching decisions. Accordingly, the study does not constitute a randomized clinical trial and does not require prospective registration in a clinical trial registry. Both groups followed the same regular endurance and technical training program, while the experimental group additionally performed SkiErg training [13].

### 2.2. Training Load Characterization

Training load characterization was implemented to ensure comparability between the control and intervention groups throughout the eight-week training period. All participants continued their regular endurance- and technique-based cross-country skiing training programs prescribed by their team coaches. These programs were maintained without any planned modifications during the study period. The intervention group additionally performed ski ergometer training sessions three times per week, whereas the control group did not engage in ski ergometer training. Apart from this addition, no differences were introduced in training frequency, volume, or intensity between groups. Athletes were instructed not to participate in any additional strength or respiratory-specific training outside the prescribed programs during the intervention period. Training intensity during ski ergometer sessions was prescribed at 80–90% of estimated maximal heart rate (HRmax), corresponding to high-intensity aerobic zones (commonly referred to as Zone 4–5). HRmax was not directly measured through maximal exercise testing; instead, it was estimated for all participants using the age-predicted formula (220-age). The same estimation procedure was applied consistently across all athletes. Heart rate was continuously monitored using chest-strap heart rate monitors to ensure adherence to the prescribed intensity zones. Although external training load was not quantified using objective metrics such as session-RPE, power output, or stroke count, training frequency, duration, and intensity zones were standardized across groups under the supervision of team coaches.

### 2.3. Population and Sample of the Study

The population of this study consisted of experienced cross-country skiers who were members of the Turkish National Cross-Country Ski Team or who competed at the regional level, were healthy, and actively continued their athletic careers. The sample group included 20 volunteer cross-country skiers who participated in the study (Experimental Group: *n* = 10, Control Group: *n* = 10).

### 2.4. Participant Flow and Attrition

A total of 20 athletes were assessed for eligibility, and all met the inclusion criteria. All participants completed the pre-test and post-test assessments, and no dropouts or exclusions occurred during the intervention period. Therefore, all enrolled participants were included in the final analysis.

### 2.5. Inclusion and Exclusion Criteria

Specific criteria were considered in the selection of participants. Accordingly, athletes who were members of the Turkish National Cross-Country Ski Team, had no musculoskeletal injuries, and voluntarily agreed to participate in the study were included. Conversely, athletes who had sustained lower or upper extremity injuries or undergone surgical procedures within the past six months, as well as those with neurological or balance-related health problems that could interfere with the study process, were excluded. In addition, participants were instructed to abstain from consuming alcohol, nicotine, or caffeine-containing substances for at least 24 h prior to testing.

### 2.6. Measurement Tool

#### 2.6.1. Assessment of Body Composition

The height of the athletes was measured using a Holtain stadiometer (Holtain Ltd., Wales, UK) with a precision of ±1 mm. Body weight, body mass index (BMI), and body fat percentage were assessed using a Tanita body composition analyzer (BC-418 model, Tanita Corp., Tokyo, Japan).

#### 2.6.2. The Ski Ergometer Test

The athletes performed their ski ergometer training sessions using a Concept2 SkiErg device (Concept2 Inc., Morrisville, VT, USA). Throughout the eight-week training period, ski ergometer workouts were conducted three times per week at an intensity corresponding to the athletes’ Zone 4–5 heart rate range (80–90% of HRmax), with each session covering a target distance of 2.5 km.

#### 2.6.3. Pulmonary Function Test

The respiratory function of the athletes was assessed using a digital spirometer (Pony FX, Cosmed, Albano Laziale, Italy). Prior to testing, athletes were instructed to fast for at least two hours, and a minimum of 15 min of rest was provided between trials. The tests were conducted with the participants seated comfortably in an upright position. During each test, the athlete’s nose was clipped, and they were instructed to seal their lips tightly around the spirometer mouthpiece to prevent air leakage. Respiratory maneuvers were performed through the mouthpiece according to standardized testing procedures. Each test was performed three times, and the best measurement value was recorded for analysis. Throughout the entire process, clear instructions were provided to the athletes, and their comfort and compliance were ensured to obtain accurate and reliable results.

#### 2.6.4. Isometric Muscle Strength Assessment

Upper-extremity isokinetic muscle strength was assessed using the ISOMED 2000 Isokinetic Dynamometer (D&R Ferstl GmbH, Hemau, Germany). Concentric elbow flexion and extension movements were evaluated at an angular velocity of 60°/s. No eccentric testing mode was applied. Prior to testing, all participants performed a standardized 15 min warm-up protocol. The first 5 min consisted of general cycling exercise on a Wattbike cycle ergometer (Wattbike Ltd., Nottingham, UK) at a workload of 70–80 W, followed by 10 min of dynamic upper-extremity mobilization and submaximal activation exercises targeting the elbow flexor and extensor muscle groups. After the warm-up, each participant was seated on the dynamometer chair with the trunk stabilized using adjustable straps across the torso and pelvis to minimize compensatory movements. The shoulder was positioned in approximately 45° of flexion in the sagittal plane, and the elbow joint axis was aligned with the dynamometer’s mechanical axis of rotation. The range of motion was set from 0° (full elbow extension) to 90° of elbow flexion. Before the recorded trials, participants completed three submaximal familiarization repetitions followed by two maximal practice trials to ensure proper technique and maximal effort. Subsequently, five maximal concentric repetitions for elbow flexion and extension were performed for each limb. A standardized rest interval of 60 s was provided between limbs. Gravity correction was applied according to the manufacturer’s guidelines prior to data collection. Strong verbal encouragement and visual feedback were provided throughout the testing procedure to ensure maximal voluntary effort.

#### 2.6.5. Outcome Definitions

The primary outcome variables were defined a priori as dominant peak power during elbow extension at 60°/s (DPPE60) and respiratory function parameters FVC and FEV_1_, based on the study hypothesis and their sport-specific relevance. All other isokinetic strength variables and additional respiratory parameters were considered secondary outcomes. Correlation analyses between dominant and nondominant limbs were conducted as exploratory analyses.

### 2.7. Statistical Analysis

Statistical analyses of the data obtained from the participants were performed using the Statistical Package for the Social Sciences (SPSS, version 22.0; IBM Corp., Armonk, NY, USA) running on Windows 11. The normality of the data distribution was assessed using the Shapiro–Wilk test and histogram plots. Data were presented as mean ± standard deviation ($\bar{X}$ ± SD). To ensure baseline equivalence between the experimental and control groups, independent samples t-tests were conducted for all baseline anthropometric and performance variables. No statistically significant differences were observed between groups at pre-test (*p* > 0.05), indicating comparable baseline characteristics. To determine the effects of the training protocol, a Two-Way Repeated Measures Analysis of Variance (ANOVA) was applied. In this analysis, the effects of time (pre-test vs. post-test), group (experimental vs. control), and the time × group interaction were evaluated. As the repeated-measures factor consisted of two time points (pre-test and post-test), the assumption of sphericity was inherently satisfied, and therefore Greenhouse–Geisser corrections were not required. For each variable, the F-value, significance level (p), and effect size (ηp^2^) were calculated. Statistical significance was set at *p* < 0.05. Partial eta squared (ηp^2^) values were interpreted as small (≥0.01), moderate (≥0.06), or large (≥0.14) effects. Measurements of isokinetic muscle strength (dominant and nondominant arms) and respiratory function parameters were analyzed separately. Additionally, Pearson correlation analysis was performed to examine the relationships between the pre- and post-test values of the dominant and nondominant limbs. The interpretation of correlation coefficients was based on Cohen’s (2013) classification [14]. Accordingly, correlation coefficients in the range of r = 0.10–0.29 were interpreted as weak, r = 0.30–0.49 as moderate, and r ≥ 0.50 as strong relationships. Effect sizes (ηp^2^) were calculated for all variables, and their magnitude was interpreted to support the practical relevance of statistically significant findings. Due to the limited availability of competitive cross-country skiers, an a priori sample size calculation was not feasible, and the sample size was determined based on practical and logistical considerations. To address concerns related to statistical power, a post hoc power analysis was conducted for the primary outcome variables. Post hoc power analysis was performed using G*Power software (version 3.1, Heinrich Heine University, Düsseldorf, Germany) based on the observed effect sizes (ηp^2^) for the primary outcomes (DPPE60 and FVC), with an alpha level set at 0.05. Given the exploratory nature of the study and the relatively small sample size, no formal correction for multiple comparisons was applied. Therefore, effect sizes were emphasized alongside *p* values, and the results—particularly those with *p* values close to the significance threshold—should be interpreted with caution.

## 3. Results

The descriptive characteristics of the participants, including age, body weight, height, and BMI, are presented in Table 1. The pre- and post-intervention changes in dominant arm isokinetic strength parameters are presented in Table 2. Significant main effects of time were observed for DPTF60, DPTAE60, DPPF60, DPPE60, and DAPF60, indicating overall improvements across the measurement period. Significant group × time interactions were detected for DPTAE60, DPPE60, and DAPE60, suggesting that the observed changes may be associated with the SkiErg training program compared to the control group. In particular, the intervention group showed a more pronounced increase in peak torque and peak power variables. No significant interaction effects were found for DPTF60, DPTE60, DPTAF60, DPPF60, or DAPF60, suggesting that changes in these variables were primarily attributable to time rather than the intervention itself.

**Table 1:** Descriptive Characteristics of the Participants.

Variable	Group	n	$\bar{X}$	SS	t	*p*
Age	CG	10	16.5	1.26	3.548	0.199
	IG	10	17.9	3.07		
	∑	20	17.2	2.39		
Body weight (kg)	CG	10	56.05	6.9	0.461	0.832
	IG	10	56.64	5.28		
	∑	20	56.34	5.99		
Height (cm)	CG	10	167.3	7.78	0.473	0.873
	IG	10	167.9	9.06		
	∑	20	167.6	8.09		
BMI (kg/m^2^)	CG	10	20	1.9	0.376	0.811
	IG	10	20.18	1.38		
	∑	20	20.09	1.62		

**Table 2:** Effects of Ski Ergometer Training on Dominant Arm Isokinetic Muscle Strength.

Test	Group	Pre-Test (Mean ± SD)	Post-Test (Mean ± SD)	Statistic (F, *p*, ηp^2^)
DPTF60	Control	44.90 ± 11.36	48.40 ± 15.47	Time: F = 4.614, *p* = 0.046, ηp^2^ = 0.204 Group: F = 1.909, *p* = 0.184, ηp^2^ = 0.096 Interaction: F = 0.128, *p* = 0.725, ηp^2^ = 0.007
	Intervention	54.32 ± 15.27	56.82 ± 16.46	
DPTE60	Control	65.99 ± 17.00	66.09 ± 19.25	Time: F = 3.710, *p* = 0.070, ηp^2^ = 0.171 Group: F = 0.522, *p* = 0.479, ηp^2^ = 0.028 Interaction: F = 3.314, *p* = 0.085, ηp^2^ = 0.155
	Intervention	70.06 ± 16.43	73.60 ± 19.31	
DPTAF60	Control	38.41 ± 10.24	38.39 ± 11.73	Time: F = 0.475, *p* = 0.500, ηp^2^ = 0.026 Group: F = 2.977, *p* = 0.102, ηp^2^ = 0.142 Interaction: F = 0.501, *p* = 0.488, ηp^2^ = 0.027
	Intervention	47.13 ± 12.98	48.61 ± 14.62	
DPTAE60	Control	58.74 ± 17.00	59.33 ± 17.24	Time: F = 8.188, *p* = 0.010, ηp^2^ = 0.313 Group: F = 1.394, *p* = 0.253, ηp^2^ = 0.072 Interaction: F = 5.862, *p* = 0.026, ηp^2^ = 0.246
	Intervention	64.60 ± 17.16	71.68 ± 18.55	
DPPF60	Control	27.80 ± 10.69	31.10 ± 12.56	Time: F = 12.571, *p* = 0.002, ηp^2^ = 0.411 Group: F = 1.168, *p* = 0.294, ηp^2^ = 0.061 Interaction: F = 0.385, *p* = 0.543, ηp^2^ = 0.021
	Intervention	33.10 ± 13.22	37.80 ± 13.97	
DPPE60	Control	48.20 ± 12.34	49.40 ± 12.20	Time: F = 33.770, *p* < 0.001, ηp^2^ = 0.652 Group: F = 1.228, *p* = 0.282, ηp^2^ = 0.064 Interaction: F = 18.590, *p* < 0.001, ηp^2^ = 0.508
	Intervention	51.60 ± 14.15	59.70 ± 16.60	
DAPF60	Control	25.70 ± 10.42	27.20 ± 9.32	Time: F = 4.426, *p* = 0.050, ηp^2^ = 0.197 Group: F = 0.979, *p* = 0.336, ηp^2^ = 0.052 Interaction: F = 0.000, *p* = 1.000, ηp^2^ = 0.000
	Intervention	30.80 ± 12.47	32.30 ± 13.80	
DAPE60	Control	45.40 ± 12.79	43.70 ± 12.79	Time: F = 2.950, *p* = 0.103, ηp^2^ = 0.141 Group: F = 1.348, *p* = 0.261, ηp^2^ = 0.070 Interaction: F = 11.466, *p* = 0.003, ηp^2^ = 0.389
	Intervention	49.10 ± 13.76	54.30 ± 16.21	

The pre- and post-intervention changes in nondominant arm isokinetic strength parameters are presented in Table 3. No significant main effects of time were observed for any of the nondominant variables (NDPTF60, NDPTE60, NDPTAF60, NDPTAE60, NDPPF60, NDPPE60, NDAPF60, and NDAPE60). Similarly, no significant main effects of group were detected for any parameter. However, significant time × group interaction effects were identified for NDPTAF60 (F = 4.786, *p* = 0.042, ηp^2^ = 0.210) and NDPPF60 (F = 8.317, *p* = 0.010, ηp^2^ = 0.316). These findings indicate differential changes between the intervention and control groups for these specific variables over the measurement period. No significant interaction effects were found for NDPTF60, NDPTE60, NDPTAE60, NDPPE60, NDAPF60, or NDAPE60.

The pre-test and post-test measurements of the control and experimental groups were compared, and the results are presented in Table 4. A significant main effect of time was observed for FVC and FEV1, with increases noted from pre- to post-test in both groups. Additionally, a significant group × time interaction was identified for FVC. For FEV1/FVC, PEF, MVV, MEP, and MIP, no significant interaction effects were detected. Similarly, no significant main effects of group were found for any of the respiratory variables.

The correlation coefficients between dominant and nondominant arm isokinetic strength variables are presented in Table 5. Strong positive correlations were observed between corresponding dominant and nondominant parameters, with correlation coefficients ranging from 0.79 to 0.92. All reported correlations were statistically significant. The highest correlation was identified between PTAE60 variables. Additionally, strong associations were observed between peak torque and peak torque average parameters across limbs.

The correlation coefficients between dominant and nondominant upper-extremity power variables are presented in Table 6. Statistically significant positive correlations were observed for all variable pairs (*p* < 0.05). Correlation coefficients ranged from 0.80 to 0.90. Significant correlations were identified between corresponding peak power flexion and extension variables of the dominant and nondominant limbs. Additionally, significant cross-parameter correlations were detected between dominant and nondominant flexion–extension combinations.

To further evaluate statistical robustness, A post hoc power analysis was conducted for the primary outcome variables using G*Power software. Based on the observed effect sizes (ηp^2^), an alpha level of 0.05, and a total sample size of 20 participants, the achieved statistical power (1 − β) was calculated as 0.91 for DPPE60 and 0.88 for FVC, indicating adequate statistical power to detect the observed effects.

## 4. Discussion

The present study examined the effects of an eight-week SkiErg-based training program on respiratory function and upper-extremity isokinetic muscle strength in cross-country skiers. The main findings indicate that several respiratory and isokinetic variables improved over time in both groups, reflecting general training-related adaptations. Importantly, a significant time × group interaction was observed for forced vital capacity (FVC) and dominant peak power during elbow extension at 60°/s (DPPE60), suggesting a potential contribution of the SkiErg intervention beyond regular training. In contrast, for most isokinetic strength variables, only a main effect of time was detected, indicating that these improvements cannot be attributed exclusively to the SkiErg program. These results highlight that SkiErg-based training may provide targeted benefits for selected respiratory and upper-extremity performance parameters, while broader strength adaptations appear to be driven primarily by overall training exposure.

Muscle strength is one of the key parameters influencing athletic performance [15]. Several factors can affect muscle strength, including muscle mass, fiber type composition, neuromuscular coordination, training status, joint angle, and the applied training methods [16]. In high-intensity sports, improving both absolute and relative strength is crucial for maintaining and enhancing performance. In combined endurance sports such as cross-country skiing, where both upper and lower limbs are actively involved, upper-body strength contributes substantially to technical efficiency and power transmission [17]. During the double-poling technique in cross-country skiing, the shoulder extensors, elbow extensors, and trunk flexors work in a synchronized manner, forming a kinetic chain that generates propulsion [18]. Therefore, the development of upper-body strength is a key determinant of success. Previous research on cross-country sit-skiing athletes has demonstrated that shoulder and elbow extension strength are strong predictors of 30 s and 3 min test performances, with isokinetic torque values measured at 60°/s explaining approximately 34–40% of performance variance [19]. This finding aligns with the present study, in which significant effects were observed in the DPPE60 test. These results may indicate a possible association between the applied training protocol and improvements in upper-extremity strength; however, due to the quasi-experimental design, causal interpretation should be made with caution. Similarly, previous studies have reported significant associations between upper-body strength and double-poling performance, emphasizing that elbow and shoulder extensor muscles play a primary role in determining poling power [20].

However, researchers have also noted that increases in isokinetic strength may not always directly translate into performance improvements, as technical proficiency and coordination mediate the conversion of strength gains into sport-specific performance [17,20]. The absence of significant interaction effects in several strength variables may also be attributed to the relatively short intervention duration, the already high training status of the athletes, and the specificity of the SkiErg movement pattern, which predominantly emphasizes extension-dominant actions rather than flexor-oriented strength adaptations. This may partly explain the absence of significant differences in certain tests. Specifically, the lack of significant group or interaction effects in DPTF60, DPTE60, DAPF60, and DAPE60 tests (*p* > 0.05) suggests that strength gains may not have occurred uniformly across all muscle groups, and that performance improvements may be limited when technical skills are not concurrently enhanced. Furthermore, Vetter et al. (2023) reported that isokinetic eccentric shoulder training improved shoulder functionality, muscle coordination, and joint stability, indicating that isokinetic training contributes not only to strength development but also to neuromuscular control and injury risk reduction [21,22]. Overall, these findings indicate that sport-specific angular velocity training may preferentially influence extension-related strength adaptations. In this context, the strength gains observed in the present study are likely related not only to muscular adaptation but also to neural adaptation and motor control improvements. Overall, the findings demonstrate that isokinetic training is effective in enhancing upper-extremity strength in cross-country skiers, particularly at angular velocities of 60°/s. This supports the notion that isokinetic training performed at sport-specific speeds yields greater functional outcomes. On the other hand, the absence of statistically significant changes in some muscle groups indicates the need to design training protocols that provide balanced stimulation across all relevant muscles.

In cross-country skiing, asymmetric muscle use is highly pronounced. Particularly during the double-poling technique, the dominant side generates the majority of the pole force, while the nondominant side plays a supporting role in balance, steering, and rhythm coordination. Therefore, strength imbalances between the dominant and nondominant arms have a direct influence on performance [23]. In the present study, the limited strength improvement observed in the nondominant arm may be a consequence of this asymmetric usage and differences in force transmission. Previous research has emphasized that bilateral strength discrepancies may affect movement economy and force transmission efficiency [19,20]. The modest improvements detected in the nondominant limb may be related to asymmetric loading patterns and incomplete bilateral neuromuscular adaptation. These findings underscore the importance of structured training approaches aimed at promoting bilateral symmetry. Isokinetic eccentric shoulder training has been reported to improve both strength development and neuromuscular coordination [21]. This suggests that incorporating bilateral load symmetry into the training protocol may promote more pronounced adaptations in the nondominant arm. Holmberg (2015) and Stög and Holmberg (2016) highlighted that the contribution of the upper limbs in cross-country skiing has progressively increased across different techniques and that bilateral coordination is critical for performance, particularly during high-intensity poling movements [18,23]. In this context, the limited improvement in nondominant arm strength observed in this study may be attributed to a technique that emphasizes the dominant side and to an incomplete development of neuromuscular coordination. Overall, the absence of significant strength gains in the nondominant arm underscores the necessity of structuring training loads to promote bilateral symmetry. The modest improvements revealed by isokinetic testing suggest that optimal balance between dominant and nondominant muscle strength may require longer training periods. In sports like cross-country skiing, which demand bilateral power generation, maintaining upper-extremity muscle balance is essential not only for performance optimization but also for reducing the risk of musculoskeletal injury.

In the literature, Holmberg and Calbet (2007) reported that the diaphragm, intercostal, and abdominal muscles are highly activated during double-poling in cross-country skiing, indicating that the discipline provides a potent stimulus for enhancing respiratory muscle endurance [18]. This physiological mechanism may explain the significant increases observed in FVC (F = 18.565, *p* < 0.001, ηp^2^ = 0.508) and FEV_1_ (F = 8.789, *p* = 0.008, ηp^2^ = 0.328) in the present study. Furthermore, the significant group × time interaction found for FVC (F = 5.014, *p* = 0.038, ηp^2^ = 0.218) suggests that SkiErg-based training may be associated with improvements in ventilatory capacity. Similarly, Vogiatzis et al. (2011) demonstrated that upper-body dominant exercises enhance respiratory muscle oxygenation and improve fatigue resistance of the ventilatory muscles [24]. This finding supports the FVC and FEV_1_ improvements observed in our study.

However, the absence of significant differences in PEF, MIP, and MEP parameters indicates that the applied protocol primarily influenced dynamic ventilation capacity rather than maximal respiratory muscle strength, suggesting that higher-resistance or longer-duration loading may be necessary for developing maximal inspiratory and expiratory pressures. Consistent with these findings, Lomax and McConnell (2003) reported that inspiratory muscle training improved ventilatory capacity but did not significantly affect peak expiratory flow (PEF) [25]. Although MVV and MEP values in the current study showed an increasing trend, the lack of statistical significance (*p* > 0.05) may be attributed to the relatively short training period. Suzuki et al. (1995) found that expiratory muscle training improved endurance, but that long-term loading was more effective in enhancing maximal pressure generation [26]. Likewise, Illi et al. (2012) emphasized that the performance benefits of respiratory muscle training primarily occur through improvements in ventilatory efficiency and fatigue resistance [27]. In this context, the concentration of improvements in ventilatory capacity parameters observed in our study indicates an enhancement in dynamic respiratory muscle endurance. SkiErg-based training appears to be a promising complementary method for improving respiratory functions in cross-country skiers. The significant increases observed in FVC and FEV_1_ parameters may reflect enhanced ventilatory capacity and respiratory muscle endurance. However, the lack of significant changes in maximal pressure parameters suggests that the inclusion of higher-resistance respiratory muscle exercises may further optimize training adaptations. The present findings are consistent with previous research [18,23], supporting the view that ventilatory efficiency—one of the key components of cross-country skiing performance—can be improved through ergometer-based training protocols.

In sports such as cross-country skiing, which require bilateral force transmission, a high level of coordination between the dominant and nondominant sides is of great importance. Upper-extremity strength balance has been reported to have a direct impact on poling power and energy efficiency in cross-country skiers [28]. In the present study, strong and positive correlations were identified between dominant and nondominant arm muscle strength measurements (r = 0.79–0.92; *p* < 0.05). The highest correlation was observed for the PTAE60 variables (r = 0.92; *p* = 0.001), followed by PTF60 (r = 0.89; *p* = 0.003), PTE60 (r = 0.87; *p* = 0.004), and PTAF60 (r = 0.84; *p* = 0.006). Although these findings indicate a high degree of association between dominant and nondominant upper-extremity strength measures, such correlations should be interpreted with caution. High inter-limb correlations may primarily reflect stable inter-individual differences in overall strength capacity rather than training-induced bilateral symmetry. Since pre-intervention correlation patterns were not directly compared with post-intervention values, it is not possible to attribute these associations specifically to the SkiErg training protocol. While bilateral strength balance is considered important for technical performance in cross-country skiing, the present findings should be regarded as descriptive rather than causal. Further longitudinal studies incorporating asymmetry indices and repeated inter-limb comparisons are required to determine whether SkiErg-based training directly influences bilateral strength adaptations.

The literature indicates that bilateral coordination and muscle symmetry in upper-extremity strength are closely associated with performance, particularly in sports characterized by repetitive and high-intensity loading, such as cross-country skiing, rowing, and swimming [29,30]. In this context, the present study revealed significant positive correlations between the dominant and nondominant sides (r = 0.80–0.90; *p* < 0.05). The highest correlation was observed between DPPF60 and NDPPF60 (r = 0.90; *p* = 0.001), indicating similar strength gains in the elbow flexor muscles of both sides. This was followed by DPPE60–NDPPE60 (r = 0.89; *p* = 0.002), DAPF60–NDAPF60 (r = 0.88; *p* = 0.002), and DAPE60–NDAPE60 (r = 0.86; *p* = 0.003). These findings may reflect similar overall strength capacities between limbs rather than direct training-induced bilateral adaptations. Aagaard et al. (2002) reported that resistance training enhances neural firing rates and helps balance bilateral force production, whereas Meylan and Malatesta (2009) demonstrated that bilateral loading promotes symmetry in motor unit activation [29,31].

These results support the high correlations observed in the present study. During the double-poling technique in cross-country skiing, synchronized force generation between both arms is essential for efficiency and movement economy. Previous studies have shown that upper-extremity strength balance is positively associated with poling power and endurance, and that bilateral coordination enhances energy efficiency [24,29]. The strong correlations found between the dominant and nondominant sides in this study may therefore reflect this technical synchronization in force production. Furthermore, Zemková et al. (2018) emphasized that bilateral strength asymmetry increases injury risk, whereas high correlation values confer advantages in both performance and protective adaptations [30]. In this regard, the high correlation levels observed in the present study suggest a well-balanced strength profile between limbs, consistent with the technical demands of cross-country skiing. Overall, these findings align with previous literature reporting that resistance and endurance training contribute to bilateral neuromuscular adaptation [23,29].

Some limitations of this study should be acknowledged. Although the sample size was relatively small in absolute numbers, it represents one of the largest and most homogeneous groups of competitive cross-country skiers available in Turkey, including national-level athletes. Additionally, maximal heart rate (HRmax) was estimated using the age-predicted formula (220-age) rather than being directly measured through maximal exercise testing. This approach may introduce inter-individual variability in the prescribed training intensity and should therefore be considered when interpreting the training stimulus. Therefore, the sample can be considered highly representative of elite cross-country skiers within the national context. Nevertheless, the controlled pre–post design without full randomization limits causal interpretation, and the correlation analyses between dominant and nondominant limbs should be regarded as exploratory rather than indicative of direct training effects. Furthermore, these correlations should be interpreted as descriptive associations rather than indicators of training-induced bilateral symmetry. Future studies should include larger multi-center samples, longer intervention periods, and randomized controlled designs to strengthen causal interpretation. Additionally, incorporating asymmetry indices, electromyographic analysis, and sport-specific performance tests may provide further insight into the bilateral adaptations induced by SkiErg-based training. Investigating the effects of combining SkiErg training with targeted respiratory muscle loading protocols may also clarify the mechanisms underlying ventilatory adaptations.

## 5. Conclusions

This study examined the effects of an eight-week SkiErg-based training program on respiratory function and upper-extremity isokinetic muscle strength in competitive cross-country skiers. The findings indicate that several performance-related variables improved over time in both groups, reflecting general training-related adaptations during the study period. Notably, SkiErg training was associated with a statistically significant interaction effect on dominant arm peak power during extension (DPPE60), as evidenced by a significant time × group interaction. In addition, a significant interaction effect was observed for forced vital capacity (FVC), suggesting that SkiErg-based training may contribute to improvements in ventilatory capacity beyond those attributable to regular training alone. For other isokinetic strength and respiratory parameters, improvements were primarily characterized by main effects of time, indicating general adaptations associated with ongoing endurance and technical training rather than a specific SkiErg-related effect. Strong correlations between dominant and nondominant upper-extremity strength measures were observed; however, these associations likely reflect stable inter-individual strength characteristics rather than training-induced bilateral symmetry. Overall, SkiErg-based training may serve as a useful complementary modality for cross-country skiers, particularly for enhancing upper-extremity power and ventilatory capacity. Nevertheless, due to the quasi-experimental design, small sample size, and absence of objective external training load measures, the findings should be interpreted with caution. Future studies employing randomized designs, larger samples, and objective load monitoring are warranted to further clarify the specific contribution of SkiErg training to performance development and its potential implications for injury prevention. From an applied perspective, coaches may consider integrating SkiErg sessions during preparatory phases to target upper-extremity power and ventilatory capacity without substantially modifying the overall endurance training structure. However, load monitoring strategies should be incorporated in future applications to ensure optimal training stimulus and recovery balance.

## Figures and Tables

**Table 3:** Effects of Ski Ergometer Training on Nondominant Arm Isokinetic Muscle Strength.

Test	Group	Pre-Test (Mean ± SD)	Post-Test (Mean ± SD)	Statistic (F, *p*, ηp^2^)
NDPTF60	Control	57.07 ± 23.89	50.05 ± 24.72	Time: F = 0.622, *p* = 0.440, ηp^2^ = 0.033 Group: F = 0.067, *p* = 0.799, ηp^2^ = 0.004 Interaction: F = 2.987, *p* = 0.101, ηp^2^ = 0.142
	Intervention	49.82 ± 17.87	52.44 ± 20.59	
NDPTE60	Control	73.00 ± 23.03	68.70 ± 24.19	Time: F = 0.742, *p* = 0.400, ηp^2^ = 0.040 Group: F = 0.036, *p* = 0.851, ηp^2^ = 0.002 Interaction: F = 2.222, *p* = 0.153, ηp^2^ = 0.110
	Intervention	72.31 ± 24.92	73.46 ± 24.55	
NDPTAF60	Control	45.20 ± 17.03	42.13 ± 20.36	Time: F = 0.373, *p* = 0.549, ηp^2^ = 0.020 Group: F = 0.099, *p* = 0.757, ηp^2^ = 0.005 Interaction: F = 4.786, *p* = 0.042, ηp^2^ = 0.210
	Intervention	40.45 ± 15.04	42.18 ± 14.52	
NDPTAE60	Control	64.31 ± 20.47	61.87 ± 23.16	Time: F = 0.060, *p* = 0.809, ηp^2^ = 0.003 Group: F = 0.047, *p* = 0.831, ηp^2^ = 0.003 Interaction: F = 1.005, *p* = 0.329, ηp^2^ = 0.053
	Intervention	64.35 ± 20.59	65.83 ± 19.86	
NDPPF60	Control	34.40 ± 15.66	29.50 ± 16.75	Time: F = 1.084, *p* = 0.311, ηp^2^ = 0.057 Group: F = 0.081, *p* = 0.779, ηp^2^ = 0.004 Interaction: F = 8.317, *p* = 0.010, ηp^2^ = 0.316
	Intervention	32.70 ± 13.94	35.00 ± 14.19	
NDPPE60	Control	51.80 ± 15.63	50.50 ± 17.14	Time: F = 0.001, *p* = 0.976, ηp^2^ = 0.000 Group: F = 0.082, *p* = 0.778, ηp^2^ = 0.005 Interaction: F = 0.589, *p* = 0.453, ηp^2^ = 0.032
	Intervention	52.80 ± 18.79	54.00 ± 19.94	
NDAPF60	Control	28.80 ± 11.68	28.70 ± 11.59	Time: F = 0.390, *p* = 0.540, ηp^2^ = 0.021 Group: F = 0.000, *p* = 0.985, ηp^2^ = 0.000 Interaction: F = 0.694, *p* = 0.416, ηp^2^ = 0.037
	Intervention	28.50 ± 12.62	29.20 ± 12.53	
NDAPE60	Control	47.70 ± 15.94	48.40 ± 17.52	Time: F = 1.185, *p* = 0.291, ηp^2^ = 0.062 Group: F = 0.138, *p* = 0.714, ηp^2^ = 0.008 Interaction: F = 0.539, *p* = 0.472, ηp^2^ = 0.029
	Intervention	49.10 ± 16.89	52.70 ± 20.16	

**Table 4:** Effects of Ski Ergometer Training on Respiratory Functions.

Test	Group	Pre-Test (Mean ± SD)	Post-Test (Mean ± SD)	Statistic (F, *p*, ηp^2^)
**FVC**	Control	4.02 ± 1.12	4.09 ± 1.17	Time: F = 18.565, *p* < 0.001, ηp^2^ = 0.508 Group: F = 0.392, *p* = 0.539, ηp^2^ = 0.021 Interaction: F = 5.014, *p* = 0.038, ηp^2^ = 0.218
	Intervention	4.27 ± 1.13	4.48 ± 1.16	
**FEV1**	Control	3.46 ± 1.06	3.55 ± 1.04	Time: F = 8.789, *p* = 0.008, ηp^2^ = 0.328 Group: F = 0.286, *p* = 0.599, ηp^2^ = 0.016 Interaction: F = 2.560, *p* = 0.127, ηp^2^ = 0.124
	Intervention	3.59 ± 0.87	3.90 ± 1.10	
**FEV1/FVC**	Control	88.40 ± 4.89	90.04 ± 5.21	Time: F = 1.411, *p* = 0.250, ηp^2^ = 0.073 Group: F = 2.128, *p* = 0.162, ηp^2^ = 0.106 Interaction: F = 0.009, *p* = 0.926, ηp^2^ = 0.000
	Intervention	85.25 ± 7.77	86.65 ± 4.66	
**PEF**	Control	6.33 ± 1.82	6.93 ± 1.58	Time: F = 2.232, *p* = 0.152, ηp^2^ = 0.110 Group: F = 0.128, *p* = 0.725, ηp^2^ = 0.007 Interaction: F = 0.015, *p* = 0.902, ηp^2^ = 0.001
	Intervention	6.59 ± 2.96	7.30 ± 2.21	
**MVV**	Control	119.10 ± 34.27	132.19 ± 36.49	Time: F = 2.475, *p* = 0.133, ηp^2^ = 0.121 Group: F = 1.184, *p* = 0.291, ηp^2^ = 0.062 Interaction: F = 0.816, *p* = 0.378, ηp^2^ = 0.043
	Intervention	141.55 ± 37.91	145.09 ± 43.48	
**MEP**	Control	132.70 ± 33.00	139.00 ± 38.34	Time: F = 0.091, *p* = 0.767, ηp^2^ = 0.005 Group: F = 0.710, *p* = 0.410, ηp^2^ = 0.038 Interaction: F = 1.112, *p* = 0.306, ηp^2^ = 0.058
	Intervention	126.40 ± 28.01	122.90 ± 24.92	
**MIP**	Control	89.60 ± 23.47	96.90 ± 19.13	Time: F = 1.652, *p* = 0.215, ηp^2^ = 0.084 Group: F = 0.387, *p* = 0.542, ηp^2^ = 0.021 Interaction: F = 0.058, *p* = 0.813, ηp^2^ = 0.003
	Intervention	96.90 ± 29.61	101.90 ± 24.85	

**Table 5:** Correlation Matrix Between Dominant and Nondominant Arm Strength Measurements-1.

Dominant	Nondominant	r	*p*
PTAE60	PTAE60	0.92	0.001
PTF60	PTF60	0.89	0.003
PTE60	PTE60	0.87	0.004
PTAF60	PTAF60	0.84	0.006
PTAE60	PTE60	0.81	0.010
PTF60	PTAF60	0.79	0.005
PTAF60	PTAE60	0.83	0.004
PTE60	PTAE60	0.88	0.002
PTAE60	PTF60	0.82	0.005
PTAF60	PTF60	0.86	0.003
PTAE60	PTAF60	0.84	0.006

**Table 6:** Correlation Matrix Between Dominant and Nondominant Arm Strength Measurements-2.

Dominant	Nondominant	r	*p*
DPPF60	NDPPF60	0.90	0.001
DPPE60	NDPPE60	0.89	0.002
DAPF60	NDAPF60	0.88	0.002
DAPE60	NDAPE60	0.86	0.003
DPPE60	NDAPE60	0.84	0.004
DPPF60	NDAPF60	0.82	0.005
DAPE60	NDPPF60	0.80	0.007
DAPF60	NDAPE60	0.83	0.004

## Data Availability

The data presented in this study are available upon reasonable request from the corresponding author.

## Abbreviations

The following abbreviations are used in this manuscript:

BMI	Body Mass Index
FVC	Forced Vital Capacity
FEV_1_	Forced Expiratory Volume in One Second
FEV_1_/FVC°	Ratio of Forced Expiratory Volume in One Second to Forced Vital Capacity
PEF	Peak Expiratory Flow
MVV	Maximal Voluntary Ventilation
MIP	Maximal Inspiratory Pressure
MEP	Maximal Expiratory Pressure
HRmax	Maximal Heart Rate
D-ND	Dominant- Nondominant
PT-PP-AP	Peak Torque- Peak Power- Average Power
F-E	Flexion; Extension
DPTF60	Dominant Peak Torque during Flexion at 60°/s
DPTE60	Dominant Peak Torque during Extension at 60°/s
DPTAF60	Dominant Average Peak Torque during Flexion at 60°/s
DPTAE60	Dominant Average Peak Torque during Extension at 60°/s
DPPF60	Dominant Peak Power during Flexion at 60°/s
DPPE60	Dominant Peak Power during Extension at 60°/s
DAPF60	Dominant Average Power during Flexion at 60°/s
DAPE60	Dominant Average Power during Extension at 60°/s
NDPTF60	Nondominant Peak Torque during Flexion at 60°/s
NDPTE60	Nondominant Peak Torque during Extension at 60°/s
NDPTAF60	Nondominant Average Peak Torque during Flexion at 60°/s
NDPTAE60	Nondominant Average Peak Torque during Extension at 60°/s
NDPPF60	Nondominant Peak Power during Flexion at 60°/s
NDPPE60	Nondominant Peak Power during Extension at 60°/s
NDAPF60	Nondominant Average Power during Flexion at 60°/s
NDAPE60	Nondominant Average Power during Extension at 60°/s
ηp^2^ °	Partial Eta Squared
